# The role of short-term volunteers in a global health capacity building effort: the Project HOPE-GEMC experience

**DOI:** 10.1186/s12245-015-0071-6

**Published:** 2015-07-21

**Authors:** Sarah D Rominski, Jamila Yakubu, Rockefeller A Oteng, Matt Peterson, Nadia Tagoe, Sue Anne Bell

**Affiliations:** Global REACH, University of Michigan Medical School, 1111 East Catherine St. 236 Victor Vaughan, Ann Arbor, MI 48109 USA; Department of Emergency Medicine, University of Michigan, 1500 E Medical Center Dr. TC-B1 354, Ann Arbor, MI 48109 USA; Project HOPE, 255 Carter Hall Lane, Millwood, VA 22646 USA; Kwame Nkrumah University of Science and Technology, Kumasi, Ghana; School of Nursing, University of Michigan, 400 North Ingalls St., Ann Arbor, MI 48109 USA

**Keywords:** Partnerships, Emergency medicine, Ghana, Global health, Capacity building

## Abstract

**Background:**

Increasingly, medical students and practicing clinicians are showing interest in traveling to low-income settings to conduct research and engage in clinical rotations. While global health activities have the potential to benefit both the individual and the host, there can be challenges. We describe one way to harmonize the desire of volunteers to have a meaningful impact on the health care delivery system in a developing country with the needs of that country.

**Methods:**

The Project Health Opportunities for People Everywhere (HOPE)-Ghana Emergency Medicine Collaborative (GEMC) Partnership has successfully integrated short-term volunteer physicians and nurses to facilitate the training of emergency medicine (EM) residents and specialist nurses in Kumasi, Ghana.

**Results:**

Since the launching of this partnership in 2011, eight physicians and 10 nurses have rotated at Komfo Anokye Teaching Hospital (KATH). The impact of these volunteers goes beyond the clinical service and supervision they provide while on the ground. They act as mentors to the trainees and assist the program leadership with teaching and assessments.

**Conclusions:**

Although generally smooth, there have been challenges, all of which have been met and are being resolved. This partnership is an example of how collaborations can harness the expertise and energy of short-term volunteers to achieve the goals of capacity building and self-sustainability.

## Background

Interest in global health experiences has been growing in recent decades. Medical students are increasingly requesting global health rotations as part of their training [1], and record numbers of medical students are traveling to low-income settings to conduct research and engage in clinical rotations [2]. Practicing clinicians have also shown interest in this type of experience. Many have engaged in medical trips to developing countries where their services are sorely needed [3]. While global health activities have the potential to benefit both the individual and the host, there can be challenges, and ethical considerations need to be examined [4]. These sorts of rotations can be a substantial burden to the host in the resource-constrained setting and can have unintended negative impacts on patients, the community, and local trainees as already scarce resources are shifted to host international visitors.

Further, the relationships between and among institutions and trainees can be unbalanced as the visitor often comes with supplies and other resources that may or may not be ideal to the local context. While supply donation seems to be positive, if those supplies are not locally available or used, these initiatives fall short. Perhaps most importantly, sustainability is not ensured with these sorts of trips [5]. Many of these global health trips or missions have been described as medical tourism, when clinicians travel to developing settings and use considerable materials, money, and logistical and human resources. There are limited data on input, process, or outcome measures of volunteer clinical missions, and thus, it is difficult to establish any long-term impact of such interventions. Further, the provision of direct clinical services by foreign volunteers does not directly build local capacity and is counterproductive to the development of sustainable health services. Considering that many countries and communities continue to repeatedly attract medical tourist visits points to a lack of local capacity building [6].

There are vast needs in developing countries in terms of health capacity building and vast resources in more developed countries. In this paper, we describe one example of a way to harmonize the desire of practicing clinicians to have a meaningful impact on the health care delivery system in a developing country with the structural needs of a country. We describe the Project HOPE-Ghana Emergency Medicine Collaborative Partnership which utilizes short-term volunteer physicians and nurses to facilitate the implementation of a residency and specialty nurse training program in Kumasi, Ghana. This approach attempts to merge the strengths of developed countries with the strengths of developing countries to address their self-identified needs. We believe this model can be useful for others interested in global health partnerships, as a way to harness the human capacity of short-term medical volunteers to build local capacity and be a part of a sustainable global health program.

### The project HOPE-Ghana emergency medicine collaborative partnership

#### Ghana emergency medicine collaborative

The Ghana Emergency Medicine Collaborative (GEMC) arose from the recognition that there was a significant need to increase human resources for health (HRH) in Ghana. Specifically, there is a need for health care workers with specialized knowledge and training in managing acutely ill and injured patients. Worldwide data indicates that injury results in 5.8 million deaths every year—32 % more than HIV, malaria, and tuberculosis combined [7]. Although this need for specialty trained health workers has been recognized, the specialty of emergency medicine (EM) is just now emerging in many developing countries, including South Africa and Ethiopia [8, 9]. Previous efforts to develop health care workers in sub-Saharan Africa have relied on the model of exporting trainees to developed countries for specialty training, with many of the trainees staying in their country of training rather than returning to their country of origin. In 2009, through discussions between the University of Michigan Department of Emergency Medicine (UMEM), Komfo Anokye Teaching Hospital (KATH), Kwame Nkrumah University of Science and Technology (KNUST), Ghana College of Physicians and Surgeons (GCPS), and the Ghana Ministry of Health (MoH), a partnership agreement was finalized to develop a 3-year postgraduate training program in emergency medicine for physicians with an aim to improve the provision of emergency care in Ghana.

To provide a trainee with the appropriate tools, training, and supervision to carry out the task of an emergency physician (EP) takes an extensive amount of clinical supervision. There is an evolved training process for EPs in the United States. This process is recognized for producing high quality clinicians, researchers, and educators. From the very beginning, GEMC was intent on creating a program that would produce not only sound clinicians but also future educators. To accomplish these goals, there was a recognized need for clinical educators.

Ideally, a program to train residents begins with a bank of fully qualified physicians locally to serve as faculty for the program. However, as this was the first program in Ghana, this was not available. GEMC approached this difficulty by securing international EM faculty (from the US and UK) to provide the educational and administrative requirements of the program, requiring a constant on-the-ground presence by US-trained and board certified EPs. The residents are recruited, supervised, and examined by the GCPS, the national body responsible for postgraduate medical training in Ghana. The graduates of the program are therefore fully certified specialist clinicians and educators. Therefore, those who are being trained by the program will take over the training of future classes of emergency medicine residents, and thus, the program will, in the future, be self-sustaining.

GEMC also recognized the need for specialty trained EM nurses to practice with the trained physicians. Working with the leadership at KATH and KNUST, a 1-year diploma program in emergency nursing (EN) for practicing nurses was developed. The program was developed in a “sandwich” format, where students are in-house at KATH for 2 weeks out of each month and at their home institutions the rest of the time. The EN diploma program encompasses the full scope of learning activities including didactic lectures, precepted clinical experience, and simulation laboratory time. The diploma, due to its unique structure, addresses a specific Ghanaian need by allowing nurses to work and train concurrently. However, a consistent challenge to making the program a success includes recruiting, funding, and supporting external faculty willing to spend 3 weeks at a time in-country. As EM is a new specialty in Ghana, there are very few in-country experts with formal EN training or experience.

As with the physician training, GEMC has committed to sending emergency nursing clinical and content experts to Ghana each month for the first 3 years of the training program. The rate of knowledge transfer, safety of patients receiving care, and overall quality of care is dependent upon quality, supervised training. Upon completion of the diploma program, nurses are qualified to take the professional examinations conducted by the Nurses and Midwives Council (NMC) of Ghana for certification.

Moving forward, the GEMC continues to collaborate with all relevant stakeholders to strengthen the partnership while promoting career advancement for graduates of the program. Additionally, the program seeks to establish new partnerships, such as the collaboration with Project HOPE, to continue providing training to residents and nurses to continue establishing the numbers of emergency medical care providers in Ghana.

#### Project HOPE

Project Health Opportunities for People Everywhere (HOPE), the People-to-People Health Foundation, was founded in 1958 with the mission to achieve sustainable advances in health care around the world by implementing health education programs and providing humanitarian assistance in areas of need. Since 1958, Project HOPE has been dedicated to developing and permanently instituting long-term solutions to pressing health problems in some of the world’s poorest countries. Today, Project HOPE’s 30+ programs are launched anywhere from the community level up to the national level and focus on five key areas: infectious disease, non-communicable disease, the health of women and children, health systems strengthening, and humanitarian assistance/disaster relief.

Project HOPE began as a volunteer-driven organization, recruiting and deploying medical volunteers to serve aboard the SS HOPE hospital ship in the 1960s and early 1970s. When the ship was retired in 1974 and HOPE’s work transitioned to land-based, volunteers played a smaller role. Since the 2004 tsunami in Southeast Asia, however, HOPE has once again used large numbers of medical volunteers in connection with their partnership with the US Navy’s Humanitarian Civic Assistance programs. Today, Project HOPE’s volunteers not only deploy with the Department of Defense humanitarian assistance/disaster relief missions but also integrate volunteers throughout the organization, cross-cutting all five key practice areas within the global health department. For Project HOPE, the GEMC project is a “volunteer-augmentation program,” where the volunteers deploy to KATH with an established, preexisting program already in place and operational. The volunteers’ roles are then focused on enhancing current activities, extending current services, and building pre-determined organizational capacity using the volunteers’ skillsets and experience.

## Methods

Personnel from Project HOPE and UM met by chance while both were attending a conference on international ethics in Gaborone, Botswana in December 2009. From preliminary conversations held at the conference, areas of intersecting interest were explored. At that time, Project HOPE was evolving their volunteer platform within the organization to begin integrating HOPE volunteers into existing programs. Emergency physicians had rotated at another Ghanaian hospital and, in a trip report, had expressed their observed need for systems changes that were not possible with short-term rotations as they did not allow adequate time for the volunteers to understand Ghanaian culture, the way the department was run, or to develop a level of trust with emergency department (ED) staff and management. Most importantly, these other hospitals were functioning not as true emergency departments with emergency physicians and nurses but rather as acute medical and surgical wards. This setup did not allow for the introduction of emergency medicine processes. Out of leadership interest at Project HOPE and a desire to integrate into ongoing, sustainable programs, through which the needed systems changes were more likely, a partnership with GEMC became attractive.

There were internal discussions at both Project HOPE and GEMC to explore the interest of both parties in joining efforts. Once it was agreed internally that a partnership appeared as though it could be mutually beneficial, there were many discussions between Project HOPE and GEMC aimed at making sure goals were agreeable to both parties.

There was a consensus within GEMC about the need for a robust selection process for interested volunteers. This process would be aimed at matching the interest of the potential volunteers with the needs of the program. It was critical to GEMC that only volunteers interested in teaching and providing supervision were accepted into the program; therefore, volunteers needed evidence of demonstrated teaching experience per their CV/employment history. Those volunteers looking for an opportunity to solely provide clinical care were not eligible to be a part of the program, and some interested volunteers were not accepted into the program because of this requirement. Matching the goals of the program with the goals of the individual volunteers—expectation management—was of paramount importance to ensure all members had a positive experience that was mutually beneficial.

Project HOPE posted a recruiting announcement for the opportunity on their website, and the EM-trained physicians or nurses who applied to the organization were directed to GEMC. Following this application, a phone call between the potential volunteer and the lead of GEMC, during which the programs aims and functions were discussed, was conducted. If the experience the volunteer was seeking matched with the GEMC program goals, they would be accepted for a rotation. GEMC maintained ultimate discretion as to who was accepted into the program. All Project HOPE volunteers are vetted and approved by both GEMC and Project HOPE. Accepted volunteers go through a rigorous screening process, to include completing a hospital credentialing packet, which requires letters of recommendation, license verification, transcripts, and letter of employment, performing a detailed background check for both legal and medical malpractice, obtaining a temporary Ghanaian nursing or medical license and registering with the Nurses and Midwifery Council of Ghana or the Medical and Dental Council of Ghana, all of which takes a minimum of 4 months from the time volunteers are accepted to the program. Recruitment criteria for nurses included a minimum of a BSN, who would only precept nursing students. A masters degree was preferred, and in this role, volunteers spent most of their time delivering didactic lectures.

In the summer of 2010, a Project HOPE program officer conducted a site visit at KATH to ensure it was a viable location for HOPE volunteers and an official memorandum of understanding (MOU) was drafted between all parties. The MOU was finalized in November 2010 and physician and nurse rotations began in April 2011.

## Results and discussion

To date, there have been 8 physicians and 10 nurses who have rotated at KATH. In total, this is almost a full year’s worth of on-the-ground faculty time by highly trained personnel. The residents at KATH have benefited greatly from the HOPE volunteers’ supervision of their clinical work. As one third year resident said, “(I) was trying to figure out what to do with a patient with pulmonary TB… The interaction taught me what to do and saved the patient’s life.” Another second year resident said, “I (believe) the Project HOPE volunteers have been very useful to us. They have been good at teaching and supportive with clinical work.” A first year resident said, “Their presence is really helpful to our residency program because we get to learn a lot any time any of them is around.”

In addition to their clinical teaching time, these volunteers have each delivered at least two lectures that have been converted to Open Education Resource (OER) format. This OER conversion means the volunteers are contributing to the growing library of materials left on the ground, and their impact is felt even after they leave Kumasi. These lectures are part of the preapproved curriculum being implemented at KATH. The impact of the Project HOPE volunteers goes beyond the clinical service and supervision they provide while on the ground. As one second year resident said, “(The Project HOPE volunteer) prompted us to always try and put ourselves in the shoes of the patients and their relatives and always treat them as we would like to be treated if we were sick.” This sentiment is one that has been repeated by several of the residents who have benefited from the work of the HOPE volunteers.

Project HOPE nursing volunteers have provided much needed support to the emergency nursing diploma program that GEMC facilitates. This collaboration has resulted in several key impacts: provision of clinical expertise, alleviating financial burdens, and contributing to faculty staffing. There are few specialty nursing training programs in Ghana, and the general nursing programs provide little education about emergency care. Many nursing students graduate with no formal education about the evaluation and management of injury or acute illness.

Project HOPE nursing volunteers fill the “expert” clinical and content gap by spending 3 weeks (the minimum time commitment) working at the bedside and precepting emergency nursing students. The sustainable training program that has been developed will be run locally within 5 years. As with the physician volunteers, the purpose of the Project HOPE nurses is not to provide clinical care but rather to teach nurse trainees clinical care.

Feedback from Project HOPE volunteers has been overwhelmingly positive. Participants were interviewed after their experience at KATH, and of those interviewed, all had a positive experience. As this was not a formal process evaluation, there was not an objective measure of satisfaction conducted, although the program reached out to all volunteers and elicited feedback. As one HOPE volunteer stated, “Overall I was very impressed with the ER Nurses at KATH. They are expected to do so much. They set up vents/bipap, care for Peds trauma patients, burns, on top of all the normal ED patients. In Red [the area of the department where the most critically injured and ill patients are cared for] everyone works great as a team in caring for the patients. It was great to be a part of their team for a few weeks. Thanks again for allowing me the opportunity to come.” The collaboration with Project HOPE has been crucial to the successful education of the first cohort of emergency nurses.

Although generally smooth, this collaboration has not been without challenges, particularly volunteer recruitment. The specific requirements for the GEMC program mean that GEMC and Project HOPE are very selective during the volunteer recruitment process. Suitable volunteers are not only required to have EM training but need to meet the necessary qualifications and experience as established by KNUST and KATH in order to teach and train EM residents and nurses.

The financial requirements for these placements are another challenge. Though some costs such as in-country flights and housing are taken up by GEMC and KATH respectively, volunteers are responsible for all other costs including international flights, visas, and credentialing fees required by Ghana’s medical and nursing regulatory bodies, which are $600 for physicians and $65 for nurses in 2015.

Providing a comprehensive orientation for volunteers has also been quite challenging. Volunteers receive an orientation document and communicate with GEMC partners via phone and e-mail prior to their departure. However, orienting the volunteers to the hospital and the program when they arrive on site has not been as efficient as it could be, as there is not a formal orientation program in place on the ground. It has also been evident that each volunteer’s needs in orientation are different, and thus, it is difficult to develop a “one size fits all” structured orientation. In addition, volunteers are expected to teach sections of the existing Ghana College of Physicians and Surgeons—accredited curriculum to physician trainees and the Ghana Nurses and Midwives Council curriculum for nurses in an unfamiliar cultural and clinical context. It can be difficult for volunteers to fully grasp the background information the trainees have received, and it can take time for each volunteer to be oriented into the clinical practice environment and what resources are available to the trainees in their practice. Further, due to the length of time volunteers are on the ground, there is a lack of continuity for the instructors. This lack of continuity is inherent in the form of the partnership as volunteers continue teaching where a previous volunteer left off. This is improving, as there are now program graduates with administrative responsibilities in the department to provide orientation and assistance to volunteers as needed.

## Conclusions

Project HOPE is a well-established organization that has a long history of international opportunities. HOPE was looking to partner with programs that had sustainable plans and models that promoted global health and was seeking to place volunteers in areas that are underserved or have a high need for skilled health workers. GEMC had a program that was built with significant Ghanaian influence and guidance for sustainability and was seeking skilled health workers and educators to advance their mission. With these goals, the two organizations filled a need for each other for a strong partnership.

Despite the few challenges in establishing and conducting this collaboration, it has been highly successful and is still ongoing. Qualitative feedback from hospital staff, leadership, and trainees has been overwhelmingly positive, indicating strong support of the partnership. There is no shortage of global health volunteer opportunities; however, we believe the partnership described above is an example of how programs can utilize the expertise and energy of short-term volunteers to impact long-term change and sustainability.

## Abbreviation

ED: emergency department
EM: emergency medicine
EN: emergency nursing
EP: emergency physician
GCPS: Ghana College of Physicians and Surgeons
GEMC: Ghana Emergency Medicine Collaborative
HRH: human resources for health
KATH: Komfo Anokye Teaching Hospital
KNUST: Kwame Nkrumah University of Science and Technology
MoH: Ministry of Health
NMC: Nurses and Midwives Council
Project HOPE: Project Health Opportunities for People Everywhere
UMEM: University of Michigan Department of Emergency Medicine
